# Peripheral Effects of FAAH Deficiency on Fuel and Energy Homeostasis: Role of Dysregulated Lysine Acetylation

**DOI:** 10.1371/journal.pone.0033717

**Published:** 2012-03-19

**Authors:** Bhavapriya Vaitheesvaran, Li Yang, Kirsten Hartil, Sherrye Glaser, Stephen Yazulla, James E. Bruce, Irwin J. Kurland

**Affiliations:** 1 Department of Medicine, Stable Isotope and Metabolomics Core Facility, Albert Einstein College of Medicine Diabetes Center, Bronx, New York, United States of America; 2 Department of Chemistry, Washington State University, Pullman, Washington, United States of America; 3 Department of Neurobiology and Behavior, Stony Brook University, Stony Brook, New York, United States of America; 4 Department of Genome Sciences, University of Washington, Seattle, Washington, United States of America; State University of Rio de Janeiro, Biomedical Center, Institute of Biology, Brazil

## Abstract

**Background:**

FAAH (fatty acid amide hydrolase), primarily expressed in the liver, hydrolyzes the endocannabinoids fatty acid ethanolamides (FAA). Human FAAH gene mutations are associated with increased body weight and obesity. In our present study, using targeted metabolite and lipid profiling, and new global acetylome profiling methodologies, we examined the role of the liver on fuel and energy homeostasis in whole body FAAH^−/−^ mice.

**Methodology/Principal Findings:**

FAAH^−/−^ mice exhibit altered energy homeostasis demonstrated by decreased oxygen consumption (Indirect calorimetry). FAAH^−/−^ mice are hyperinsulinemic and have adipose, skeletal and hepatic insulin resistance as indicated by stable isotope phenotyping (SIPHEN). Fed state skeletal muscle and liver triglyceride levels was increased 2–3 fold, while glycogen was decreased 42% and 57% respectively. Hepatic cholesterol synthesis was decreased 22% in FAAH^−/−^ mice. Dysregulated hepatic FAAH^−/−^ lysine acetylation was consistent with their metabolite profiling. Fasted to fed increases in hepatic FAAH^−/−^ acetyl-CoA (85%, p<0.01) corresponded to similar increases in citrate levels (45%). Altered FAAH^−/−^ mitochondrial malate dehydrogenase (MDH2) acetylation, which can affect the malate aspartate shuttle, was consistent with our observation of a 25% decrease in fed malate and aspartate levels. Decreased fasted but not fed dihydroxyacetone-P and glycerol-3-P levels in FAAH^−/−^ mice was consistent with a compensating contribution from decreased acetylation of fed FAAH^−/−^ aldolase B. Fed FAAH^−/−^ alcohol dehydrogenase (ADH) acetylation was also decreased.

**Conclusions/Significance:**

Whole body FAAH deletion contributes to a pre-diabetic phenotype by mechanisms resulting in impairment of hepatic glucose and lipid metabolism. FAAH^−/−^ mice had altered hepatic lysine acetylation, the pattern sharing similarities with acetylation changes reported with chronic alcohol treatment. Dysregulated hepatic lysine acetylation seen with impaired FAA hydrolysis could support the liver's role in fostering the pre-diabetic state, and may reflect part of the mechanism underlying the hepatic effects of endocannabinoids in alcoholic liver disease mouse models.

## Introduction

Obesity, now recognized as a chronic disease, is the second leading cause of preventable death. In 2008 the World Health Organization estimated 1.5 billion adults, 20 and older, were overweight. Of these over 200 million men and nearly 300 million women were obese (http://www.who.int/mediacentre/factsheets/fs311/en/) [1]. It is well established that obesity is a major risk factor for the development of Type II diabetes (‘diabesity’) [2].

The endogenous cannabinoid system, is comprised of i) endogenously produced ligands, the endocannabinoids, ii) cannabinoid receptors and iii) cannabinoid metabolizing enzymes [3], plays a crucial role in controlling a diversity of physiological and behavioral processes including those involved in energy homeostasis [4], [5], [6]. Several studies have shown that the endocannabinoid system is dysregulated [7], [8], [9], [10] and activated in peripheral tissues [11], [12], [13] during obesity.

The fatty acid amides (FAA), anandamide and 2-arachidonoyl glycerol (2-AG) are the most widely studied cannabinoid ligands [14], [15], [16]. These lipids are present throughout the body and their levels are finely regulated by the balance between synthesis and inactivation [17]. FAA hydrolase (FAAH) is the main FAA degrading enzyme, primarily acting on anandamide (AEA), an endogenous ligand of CB_1_ cannabinoid receptors, and oleoylethanolamide (OEA), which binds to peroxisome proliferator-activated receptors-α to reduce food intake and promote lipolysis [5]. Studies have shown that a missense polymorphism in the FAAH gene is associated with severe obesity (BMI≥40), along with increased plasma levels of anandamide (AEA), and related N-acylethanolamines [18], [19]. However, the precise role of FAAH in the regulation of energy expenditure and fuel homeostasis is not well understood. Tourino et al. showed that on a high-fat diet, FAAH^−/−^ mice had elevated hypothalamic, hepatic and small intestinal AEA and OEA levels [5]. Despite comparable caloric intake, these high-fat fed, FAAH^−/−^ mice showed obesity and elevated ad-lib glucose and insulin levels implying dysregulation of energy storage and/or expenditure [5].

In our present study, the contributions of liver FAAH absence to the effects of whole body FAAH deletion on fuel and energy homeostasis was examined utilizing fluxomics, targeted metabolite and lipid profiling, and in particular, a new global acetylome profiling method. Stable isotope flux phenotyping revealed that FAAH^−/−^ mice displayed hepatic, skeletal and adipose insulin resistance. Label-free quantitation of the hepatic acetylome under different nutritional states, demonstrated that FAAH^−/−^ mice exhibit dysregulated lysine acetylation of enzymes in key metabolic pathways. The functionality of altered acetylation of specific proteins was further assessed by metabolite analyses.

Our study suggests that FAAH^−/−^ mice are a model of the pre-diabetic state, having adipose, skeletal and hepatic insulin resistance, preserved skeletal muscle fuel switching, and unsuppressed hepatic glucose production (HGP) with impaired glucose tolerance. Liver FAAH absence contributes to the pre-diabetic phenotype of FAAH^−/−^ mice by mechanisms, at least in part, dependent on dysregulated lysine acetylation, resulting in impairment of hepatic glucose and lipid metabolism.

## Results

### The Obese Pre-Diabetic Phenotype of FAAH^−/−^ Mice

FAAH^−/−^ mice on a regular chow were significantly (1.25-fold) heavier than age matched wild-types (Table 1). Increased body weight could be attributed, in part, to increased food intake by FAAH^−/−^ mice, which consumed 26% more food/day compared to wild-types (not different when normalized to body weight).

**Table 1:** General body composition, basal glucose and insulin.

	Wild-type(n = 5)	FAAH^−/−^(n = 5)
Body weight	28.72±0.64	34.95±3.36**
Food intake (gms/day)	3.5±0.4	4.41±0.62*
% Fat Mass	8.8±0.2 (n = 6)	13.7±1** (n = 9)
% Lean Mass	86.6±0.4 (n = 6)	82.2±1* (n = 9)
Basal glucose (15 h fast mg/dl)	148±5.1	134±13.40
Basal insulin (15 h fast ng/ml)	0.474±0.13	1.351±0.13**

Data are mean±SEM.

* p<0.05,

** p<0.01 by Student's t-test for wild-type vs. FAAH^−/−^ mice.

Body composition analysis revealed that FAAH^−/−^ mice had significantly higher fat mass and lower lean mass (Table 1). After a 15 h overnight fast, FAAH^−/−^ mice displayed nearly three-fold higher basal insulin levels compared to wild-types. Fasting basal glucose was not different between the two groups. The obesity and hyperinsulinemia with normal glucose levels observed in FAAH^−/−^ mice is similar to that reported in pre-diabetic individuals with whole body insulin resistance. Both insulin resistance and metabolic inflexibility have been described as co-incident in obesity [20], [21], therefore we examined metabolic inflexibility and energy expenditure in FAAH^−/−^ mice by indirect calorimetry.

### FAAH^−/−^ Mice are Metabolically Flexible but Exhibit Decreased Energy Expenditure

**Figure 1:**
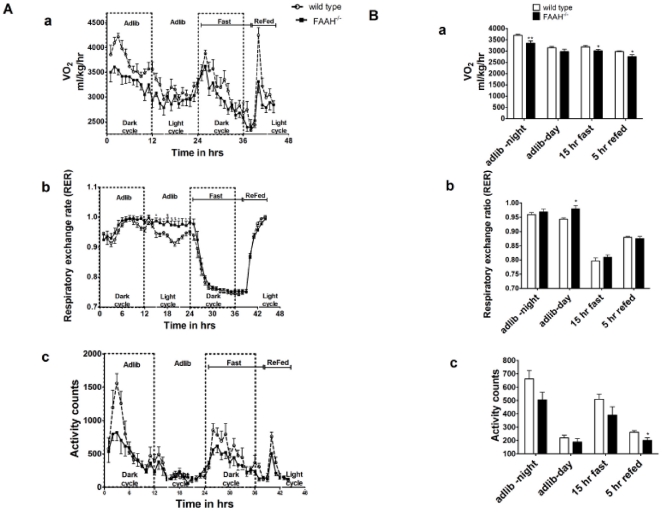
Indirect calorimetry of FAAH^−/−^ and wild-type mice. A) hr-hr B) 12 hr average. a) Oxygen consumption (VO2), b) Respiratory exchange ratio (RER) and c) Activity during the diurnal cycle and fasted to fed transitions. Day (light cycle) and night (dark cycle) 12 hours, (over) night fast −15 h, day re-fed −5 h in duration. n = 8, data are mean ± SEM, *p<0.05, **p<0.01 by Student's t-test.

To determine the effect of FAAH deletion on energy expenditure, FAAH^−/−^ and wild-type mice were subjected to indirect calorimetry. Figure 1A (h-h) and 1B (12 h average). Oxygen consumption (VO_2_), RER and activity were measured during the ad-lib state (light/dark cycles) and during a 12 h overnight fast followed by 5 h of re-feeding. FAAH^−/−^ mice displayed a significant decrease in VO2 during all conditions, even when normalized to lean body mass (data not shown).

RER was similar between FAAH^−/−^ and wild-type mice during ad-lib dark cycle, and during fasting/re-feeding, implying preservation of skeletal muscle fuel utilization/flexibility. During the ad-lib light cycle however, RER was higher in FAAH^−/−^ mice compared to wild-types (Fig. 1A–b). This suggests that FAAH^−/−^ mice were eating during this time, consistent with increased food intake reported above. Ambulatory activity was significantly lower for the FAAH^−/−^ mice during the 5 hr of re-feeding following the fast (Fig. 1B–c), but was overall similar between FAAH^−/−^ and wild-type mice (Fig. 1A–c).

### FAAH^−/−^ Mice have Dysregulated Lipid and Glycogen Levels

**Figure 2:**
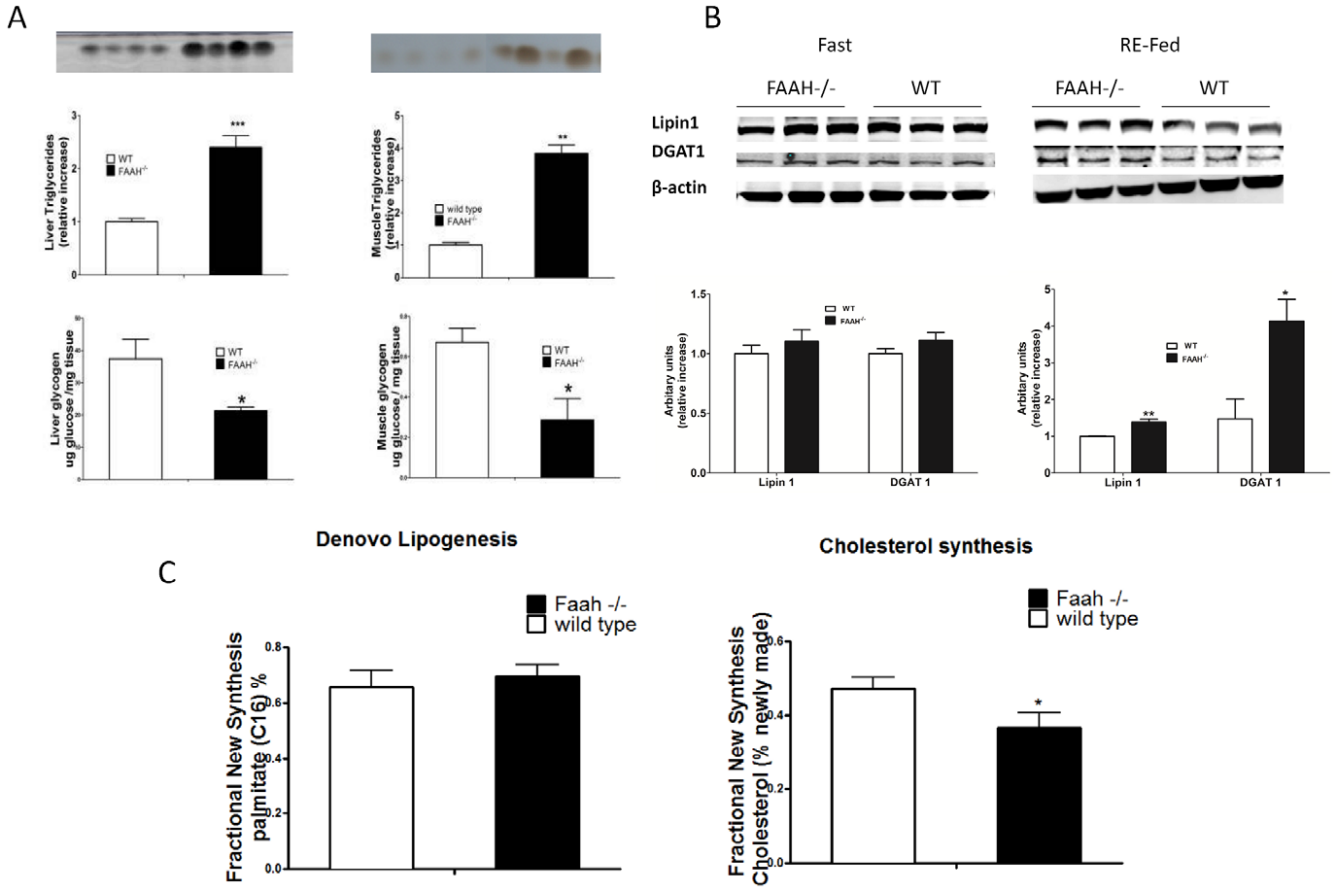
FAAH deficiency affects fuel storage. a. Fed triglycerides and glycogen of liver and skeletal muscle. Upper panel shows thin layer chromatography (TLC) for hepatic and intra-muscular triglycerides with corresponding densitometry. The lower panel shows the amount of glycogen in the same tissues. Data are mean ± SEM, n = 4, *p<0.05, **p<0.01, ***p<0.001 FAAH^−/−^ vs. wild-type by Student's t-test. b. Immunoblot analysis for Lipin 1 and DGAT 1 in overnight fasted (18 h) and 5 h re-fed liver (Top). Quantification normalized by actin content and arbitary units expressed relative to wild-type (Bottom). n = 4, data are mean ± SEM. *p<0.05, **p<0.01 by Student's t-test. *c.* De novo lipogenesis and Cholesterol synthesis. Synthesis rates measured in fed FAAH^−/−^ mice vs. wild-type mice over a 10 day period. Data are mean ± SEM, n = 6, *p<0.05 by Student's t-test.

Following an 18 h fast hepatic triglyceride (TAG) levels were similar in FAAH^−/−^ and wild-type mice (data not shown). In contrast, re-fed FAAH^−/−^ mice had a 2-fold increase in both hepatic and skeletal muscle TAGs (TLC, Fig. 2a). The hepatic and muscle TAGs were also quantified per mg tissue, and given in Figure S3. Interestingly, fed FAAH^−/−^ hepatic and skeletal muscle glycogen levels were significantly (p<0.05) decreased compared to wild-types (Fig. 2a). Hepatic *de novo* lipogenesis was not different between FAAH^−/−^ and wild-type mice, while cholesterol synthesis was significantly lower in FAAH^−/−^ mice (Fig. 2c). Fasted/fed levels of key metabolic proteins involved in hepatic fuel switching showed no differences [fatty acid synthase (FAS), ATP citrate lyase (ACL), glucose-6-phosphate dehydrogenase (G6PDH), Rheb, 6-phosphofructo-2kinase (6-PF2K-B), glucokinase (GCK), AMPK and acetyl-CoA carboxylase (ACC), Figure S1]. The normal levels of these proteins may reflect the pre-diabetic state, preserving control of lipogenesis at the expense of hyperinsulinemia.

**Figure 3:**
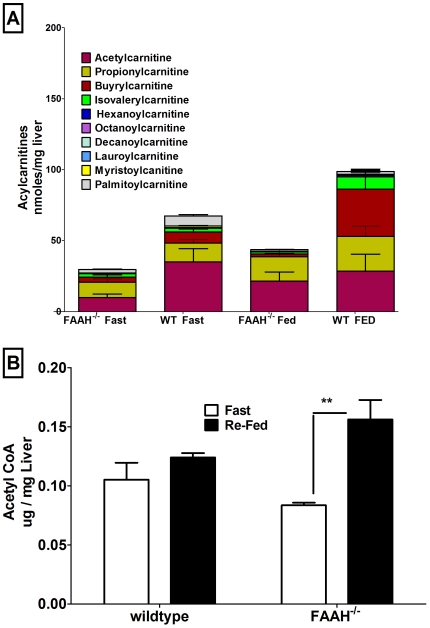
FAAH deficiency affects disposition of hepatic acyl carnitine and acetyl-CoA. a. Hepatic acyl carnitine profile in 18 h fasted and 5 h re-fed mice. Data are mean ± SEM, n = 4 for FAAH^−/−^ vs wild-type mice. Non-repeated measures 2-way ANOVA was done to calculate statistical significance. P<0.001 for both row and columns factors. Bonferroni post-tests showed P<0.001 for FAAH^−/−^ acetylcarnitine. b. Hepatic acetyl-CoA levels in 18 h fasted and 5 h re-fed mice. Data are mean ± SEM, n = 4, **p<0.01 by Student's t-test for FAAH^−/−^ vs wild-type mice.

Acylcarnitines, prerequisites in the oxidation of fatty acids, were overall significantly decreased in both fasted and fed FAAH^−/−^ livers (Fig. 3a). Despite decreased hepatic acylcarnitines, which imply increased fatty acid oxidation (FAO), FAAH^−/−^ livers accumulated TAG. Increased TAG accumulation may reflect increased re-esterification, due to increased adipose lipolysis/plasma free fatty acids (NEFAs) in the fasted state (see below), indicated by increased levels of lipin 1 and DGAT 1 in the fed state (Fig. 2b)

### FAAH Deficiency Results in Adipose Tissue Insulin Resistance

**Figure 4:**
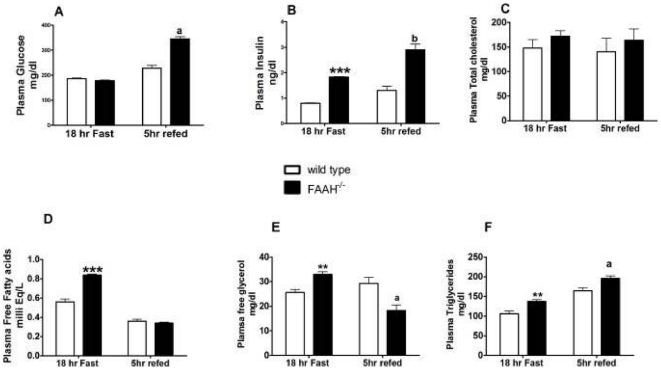
FAAH deficiency causes dyslipidemia. Fasted/re-fed plasma glucose and lipid profile for the FAAH^−/−^ and wild-type mice. Plasma samples used were from [U-^13^C_6_] glucose (18 h fast) and [2-^13^C] glycerol (5 h re-feed) tracer infusion experiments. n = 5, data are mean ± SEM. **p<0.01, ***p<0.001 FAAH^−/−^ vs. wild-type (fasted) and a p<0.05, b p<0.01, FAAH^−/−^ vs. wild-type (fed) by Student's t-test.

The fasted/re-fed plasma profile is summarized in Figure 4. 18 h fasted FAAH^−/−^ mice had significantly higher insulin, NEFAs, free glycerol and TAG levels compared to wild-type mice. Elevated lipids, together with high plasma insulin levels, suggest unsuppressed lipolysis, indicative of adipose tissue insulin resistance.

Upon re-feeding, FAAH^−/−^ plasma glucose, insulin and TAGs were significantly higher compared to wild-type mice. Free glycerol was lower and NEFAs were not different in FAAH^−/−^ mice compared to wild-types. Plasma total cholesterol remained unchanged during both fasted and re-fed states between the two groups of mice.

**Figure 5:**
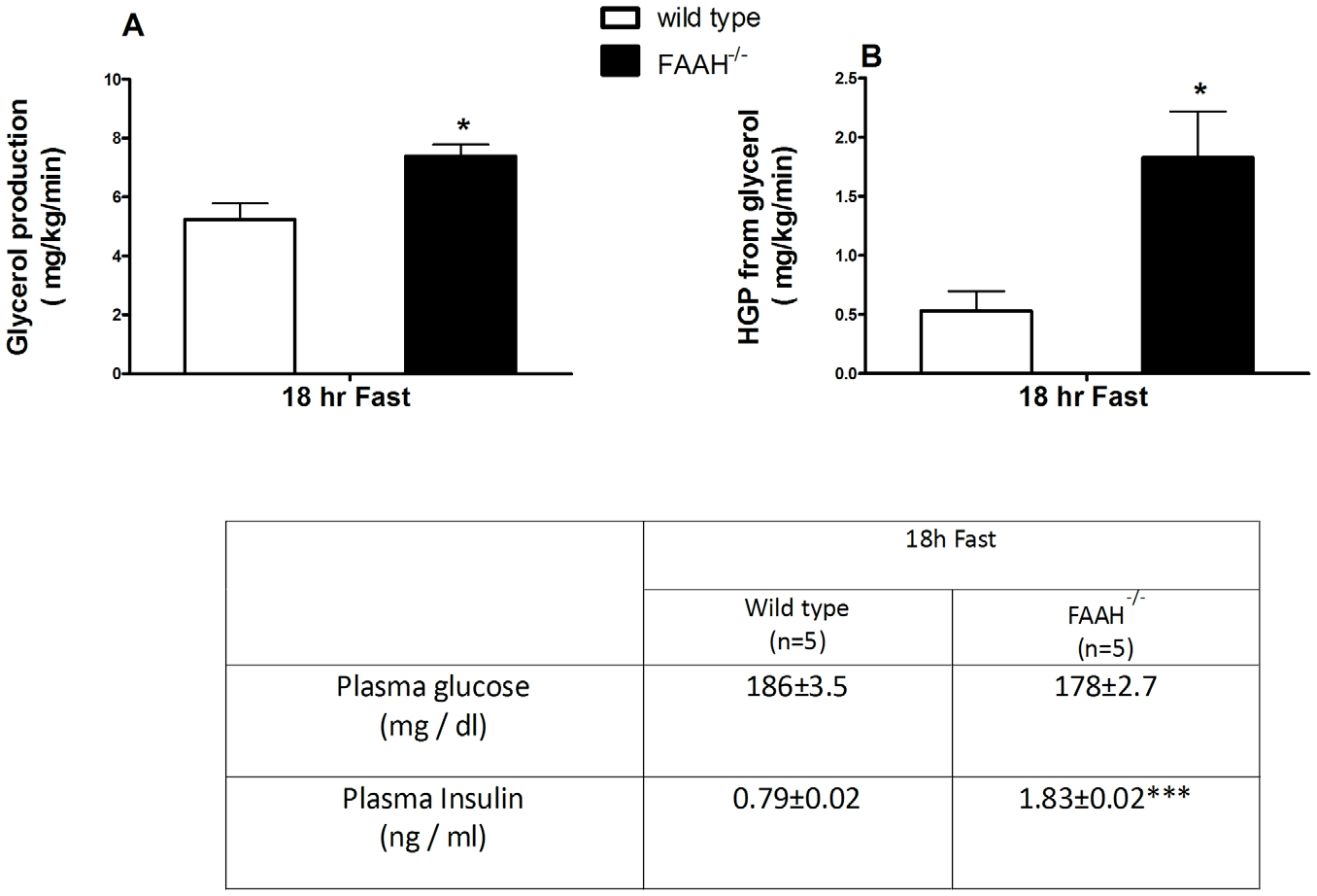
FAAH deficiency causes hepatic and adipose insulin resistance. Glycerol production, and hepatic glucose production from glycerol, assessed using a [2-^13^C] glycerol infusion administered by Alza miniosmotic pump. Glycerol production represents mainly in vivo lipolysis, and was measured after 18 h of overnight fast. Fasted plasma glucose and insulin levels are given in the table. Glycerol production rate is expressed in terms of mg produced/kg of body weight/minute. n = 5, data are mean ± SEM. *p<0.05, ***p<0.001 wild-type vs. FAAH^−/−^ mice by Student's t-test.

Adipose tissue lipolysis was assessed by measuring glycerol production using 2-^13^C glycerol infusions (Fig. 5). After an 18 h fast, despite increased insulin levels, FAAH^−/−^ mice had a 40% increase in glycerol production (lipolysis), and a 1.5-fold increase in hepatic glucose production (HGP) from glycerol compared to wild-types (Fig. 5A and 5B respectively). Glycerol production and HGP from glycerol following re-feeding were similar between FAAH^−/−^ and wild-type mice (data not shown). The decreased triose-P metabolites in the fasted state of FAAH−/− mice (Table 2) also support the re-direction of triose-P intermediates to HGP.

**Table 2:** Fasted/Re-fed hepatic triose-p metabolites profile.

nano moles/mg liver	18 hr Fast		5 hr Re-feed	
	Wild-typen = 4	FAAH^−/−^n = 4	Wild-typen = 4	FAAH^−/−^n = 4
**Glycerol 3 po4**	6.235±0.31	4.375±0.63*	6.801±1.11	4.606±0.93
**Glyceraldehyde-3-P**	0.246±0.08	0.171±0.07	0.191±0.03	0.170±0.03
**Dihdroxyacetone-P**	1.596±0.07	1.106±0.16*	1.827±0.34	1.167±0.23

Values are data±SEM,

* p<0.05, FAAH^−/−^ vs. wild-type mice by Student's t-test.

### FAAH^−/−^ Deficiency Results in Hepatic Insulin Resistance

**Figure 6:**
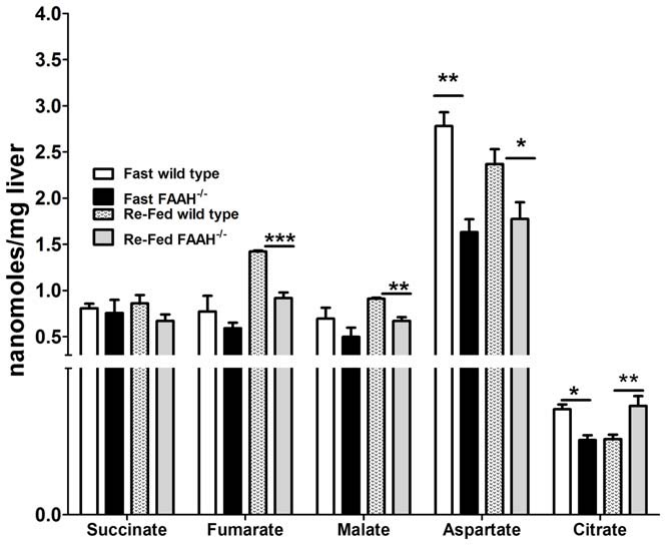
FAAH deficiency causes fasted/re-fed dysregulation of hepatic TCA intermediates. Hepatic TCA intermediates in 18 h fasted and 5 h re-fed mice. Data are mean ± SEM, n = 4, *p<0.05, **p<0.01, ***p<0.001 by Student's t-test in FAAH^−/−^ mice vs. wild-type mice respectively.

The unsuppressed HGP from glycerol, despite basal hyperinsulinemia, seen in FAAH^−/−^ mice suggested hepatic insulin resistance. This was confirmed by measuring HGP during an 18 h fast using U-^13^C glucose infusions (Table 3). Despite 2-fold higher basal insulin levels in FAAH^−/−^ mice, HGP and plasma glucose levels were comparable to wild-types. FAAH^−/−^ livers failed to suppress HGP, despite elevated plasma insulin. This indicates a significant degree of hepatic insulin resistance in FAAH^−/−^ mice for these constant glucose infusion conditions. Figure 6 shows alterations in hepatic TCA cycle and glycolytic intermediates for the fasted and/or fed states, another manifestation of hepatic insulin resistance in FAAH^−/−^ mice. Fumarate, malate and aspartate were decreased, and citrate increased, in the fed state of FAAH^−/−^ mice, while in the fasted state, aspartate, citrate, glycerol-3-P and dihydroxyacetone-P were decreased for FAAH^−/−^ mice, suggesting alterations in the malate-aspartate shuttle, and glycolysis.

**Table 3:** Plasma glucose and insulin during [U-^13^C_6_] glucose pump assessment following an 18 hr fast.

	Wild-type(n = 5)	FAAH^−/−^(n = 5)
Plasma glucose (mg/dl)	186±3.5	178±2.7
Plasma insulin (ng/ml)	0.79±0.02	1.83±0.02***
Hepatic Glucose Production (mg/kg/min)	38.5±5.5	31.6±2.84
Clearance (ml/min)	20.8±3.2	17.78±1.7

Data are mean ± SEM.

*** P<0.001, wild-type vs. FAAH^−/−^ mice by Student's t-test.

### FAAH^−/−^ mice have Preserved Peripheral Glucose Disposal

**Figure 7:**
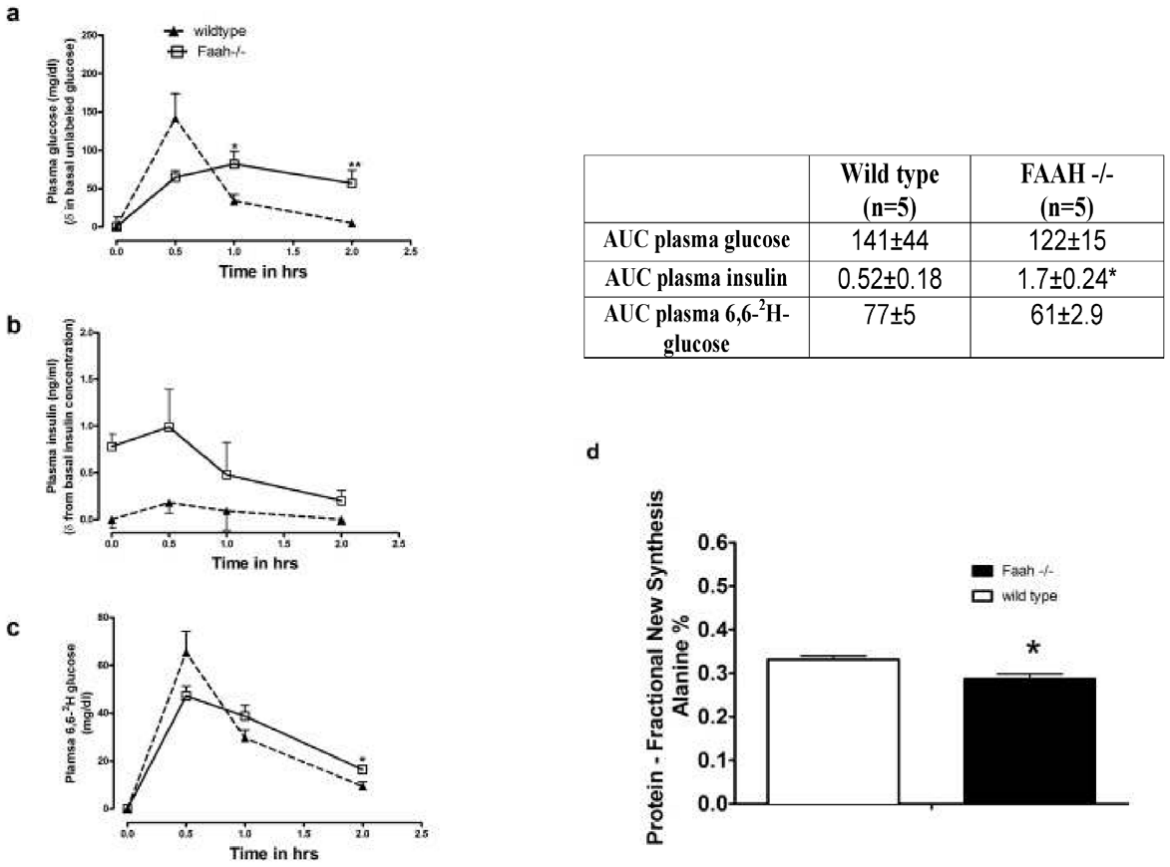
FAAH deficiency causes skeletal muscle insulin resistance. Glucose disposal measured during the Stable isotope Glucose Tolerance Test (SipGTT) for chow fed, overnight fasted, FAA^H−/−^ vs. wild-type mice. Time courses of plasma glucose (a), insulin (b) and [6, 6-2H2]-glucose (c) normalized to wild-type basal levels are shown (Left). Each point shown represents the mean ± SEM, n = 5. Integrated responses for the areas under the curve (AUC) are presented in the table shown (Top right). *p<0.05, **p<0.01 for (FAA^H−/−^ vs. wild-type). Muscle (quadriceps) protein synthesis in FAA^H−/−^ and wild-type mice (Bottom right) (d). Protein synthesis is represented as % newly made alanine made over the entire 10 day study. Data are mean ± SEM. *p<0.05, comparing n = 6 for wild-type and FAA^H−/−^ mice by Student's t-test.

Figure 7 shows the time course of the change in (a) plasma total glucose, (b) insulin, and (c) [6, 6-^2^H]-glucose during a stable isotope labeled GTT (SipGTT). Integrative responses are summarized in the inset table on right. The area under the curve (AUC) for plasma glucose and [6, 6-^2^H] curve was not different between wild-type and FAAH^−/−^ mice indicating no change in dynamic glucose disposal. However, hyperinsulinemia in FAAH^−/−^ mice was evident indicated by their AUC for plasma insulin which was 3 times higher compared to wild-type mice. Total plasma glucose was elevated in the latter half of the GTT, consistent with non-suppressible HGP.

Skeletal muscle protein synthesis was significantly decreased in FAAH^−/−^ mice (Fig. 7d), which, along with decreased fed muscle glycogen stores, is also an indication of skeletal muscle insulin resistance.

### FAAH Deficiency Results in Dysregulation of the Hepatic Acetylome

The changes from fasted to fed hepatic acetyl-CoA levels were significant for FAAH^−/−^ mice, in contrast to wild-type mice (Fig. 3b), suggesting an overall increase in acetyl-CoA levels in response to re-feeding in the absence of FAAH. Dysregulation of acetyl-CoA levels could affect feedback regulation of the metabolic acetylome [22], supported here by our analysis of the 18 h fasted/5 h re-fed hepatic proteome for lysine acetylation. A global representation of dysregulated lysine acetylation in FAAH^−/−^ livers using immunoprecipitation (IP) with an anti-acetylated lysine antibody followed by western blot analysis is shown in Figure S4a. Western analysis of the anti-acetylated lysine IP for two important acetylation target proteins (alcohol and malate dehydrogenase) was performed to demonstrate differential fasting to re-fed regulation of acetylation (Fig. S4b). Since all the acetylated sites on proteins may not even be accessible to the anti-acetylated lysine antibody for IP, label-free proteomics was performed.

**Figure 8:**
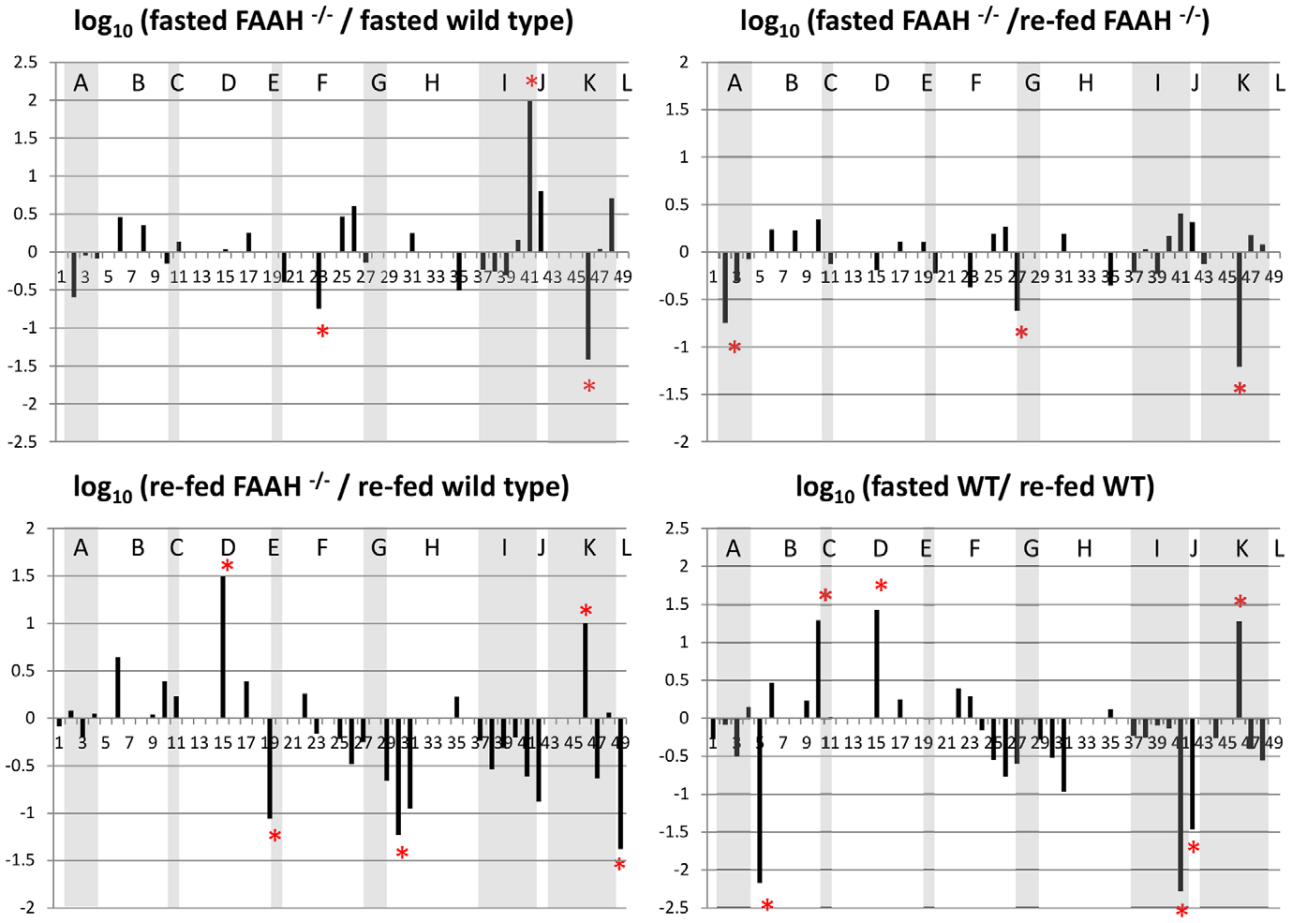
FAAH deficiency causes dyregulates the hepatic acetylome. Log_10_ ratios of the fasted and re-fed, wild-type and FAAH^−/−^ liver acetylated peptides. The top 5% dramatically changed acetylated peptides are marked with red *. Acetylated peptides from the same proteins are grouped and shaded with gray background color. The 12 proteins are indicated with single letters on top of each group of peptides in the figure and their names are as follows: A. Superoxide dismutase; B. Aspartate aminotransferase; C. T-lymphoma invasion and metastasis-inducing protein 1; D. ATP synthase coupling factor 6; E. Fructose-bisphosphate aldolase B; F. Acetyl-CoA acetyltransferase; G. GTP:AMP phosphotransferase mitochondrial; H. 3-ketoacyl-CoA thiolase; I. ATP synthase D chain; J. Phosphoglycerate mutase 2; K. Malate dehydrogenase; L. Alcohol dehydrogenase.

In total we identify and report 217 peptides from 95 proteins (Table S1). Validated peptides with a common sequence but modifications at different sites are treated as different entries in the table. An index number was given to each entry. Multiple acetylated peptides from the same protein were grouped in the list, so they have neighboring index numbers. The list of largely altered acetylated peptides, their sequences, protein origins with their index values are summarized in Table S2. In total, 49 acetylated peptides from 12 different proteins that were altered are presented. Table S3 compares and summarizes the acetylated peptides and corresponding proteins that showed more than a four-fold change between 18 h fast/5 h fed FAAH^−/−^ and wild-type mice. Figure 8 shows the log_10_ ratios of the EIC (extracted ion chromatogram) peak areas of the largely changed peptides, comparing between the wild-type and FAAH^−/−^ groups, as well as their feeding conditions. Log_10_ ratio higher than or lower than 0 indicates that abundance of the acetylated peptide either is increased or decreased respectively. All signals were normalized to the most abundant acetylated peptide observed, and the ratios were calculated from the average EIC area values from triplicate LC-MS/MS experiments. For cases where several acetylated peptides are originated from the same protein sequence, the results were grouped and shaded with gray background color. The 12 proteins correspond to these groups of peptides are indicated with single letters on top of each group. Distribution of the Log_10_ values for all the quantified acetylated peptides from FAAH^−/−^ vs. wild type mice observed under fasted and re-fed conditions are illustrated in Figure S2.

Predominately mitochondrial proteins in addition to glycolytic enzymes were affected by the fasted/re-fed dysregulation in lysine acetylation in FAAH^−/−^ liver. Fasted FAAH^−/−^ showed an increase in the acetylation of ATP synthase subunit d, and a decrease in the acetylation of acetyl-CoA acetyltransferase (ACAT1) and malate dehydrogenase (MDH2) compared to wild-type mice (Table S3). Fed FAAH^−/−^ mice showed an increase in the acetylation of ATP coupling factor 6 and MDH2, and a decrease in the acetylation of fructose-bisphosphate aldolase B, 3-ketoacyl-CoA thiolase and alcohol dehydrogenase compared to wild-type mice.

Changes in hepatic metabolic flexibility/fuel switching may be reflected by the differences seen in fasted/re-fed acetylation. The re-fed versus fasted FAAH^−/−^ mice had increases in acetylation for MDH2, adenylate kinase and superoxide dismutase. In contrast, the re-fed versus fasted wild-type mice had increases in acetylation for ATP synthase subunit d, aspartate aminotransferase and phosphoglucomutase 2 and decreases in acetylation for MDH2, ATP synthase-coupling factor 6, and Tiam1.

The significance of changes in acetylation we observed is emphasized by the metabolite changes measured (Fig. 6). Citrate which was decreased in fasted FAAH^−/−^ mice, increased significantly upon re-feeding, reflecting changes in acetyl-CoA levels (Fig. 3b). Mitochondrial MDH2, was hypoacetylated in fasted FAAH^−/−^ livers, and hyperacetylated in fed FAAH^−/−^ livers. While fasted fumarate and malate levels were comparable between the groups, they were significantly decreased in fed FAAH^−/−^ mice consistent with the observed dysregulated MDH2 acetylation. Aspartate aminotransferase (AST), hyperacetylated in wild-type mice during the re-fed/fasted transition had no change for FAAH ^−/−^ mice during such transition. Consistently, compared to the wild-types, FAAH^−/−^ aspartate levels were decreased in both states supporting altered AST acetylation.

## Discussion

FAAH^−/−^ mice represent a powerful model system to examine the central and peripheral consequences of constitutive inactivation of FAA catabolism [23], [24]. FAAs such as AEA play a crucial role in controlling hunger and development of obesity [25], [26]. Weight gain in FAAH^−/−^ mice may be secondary to over-eating, in addition to a direct effect of AEA on adipocyte differentiation [27], [28], [29].

In our present study we have examined the metabolic effect of FAAH gene deletion on fuel switching and energy homeostasis. Even though liver is the primary insulin sensitive peripheral site for FAAH expression, SIPHEN assessment concluded that FAAH deficiency results in whole body insulin resistance, demonstrating a well defined pre-diabetic phenotype. Using SIPHEN, we recently established the MKR mouse as a model of Type II DM and metabolic inflexibility [30]. In this study, we now demonstrate that the FAAH^−/−^ mouse may be an important pre-Type II DM model. We show that whole body FAAH deletion in mice mimics several metabolic aspects of pre-diabetes including impaired fuel utilization, hyperinsulinemia, and hepatic, skeletal muscle and adipose tissue insulin resistance.

Tourino et al [5], showed that although overall daily food intake was comparable between FAAH^−/−^ and wild-type mice, caloric intake during dark (∼re-fed) and light (∼semi-fasted) conditions were significantly different. In our study, by combining calorimetry with metabolic assessments of tissue fuel stores (cholesterol and fatty acid synthesis, TAG and glycogen levels) after a defined overnight fast, with or without re-feeding, a more definite assessment of the effects of FAAH deficiency on overall fuel and energy homeostasis could be made. Consistent with the ad lib model of Taurino et al, fed intra-muscular and hepatic TAG levels were increased in FAAH^−/−^ mice compared to wild-types. But TAG levels (liver and muscle) were normal in the fasted state. Moreover, observations of decreased fed skeletal muscle and hepatic glycogen stores, together with decreased skeletal muscle protein synthesis despite hyperinsulinemia in our study, establishes, and is consistent with, skeletal muscle and hepatic insulin resistance.

Skeletal muscle fuel switching is preserved in FAAH^−/−^ mice, despite increased TAG stores. The preservation of the respiratory exchange ratio (RER) between fasting and re-feeding (Fig. 1), implies preservation of skeletal muscle glucose disposal. HGP equals basal glucose disposal in the fasted state [30], [31], and was unchanged between FAAH^−/−^ and wild-type mice. This is probably due, in part, to hyperinsulinemia, preserving skeletal muscle metabolic flexibility at the cost of insulin resistance. The liver is insulin resistant to the extent that only lipogenesis is preserved, as fed glycogen is decreased, and basal HGP is unsuppressed. As shown in Fig. 7c, [6,6-^2^H] glucose disposal is preserved, but plasma glucose levels are elevated in the latter half of the GTT, suggesting increased post-prandial HGP. Hyperinsulinemia is severe enough that secondary adipose insulin resistance occurs, as evidenced by an increased rate of basal lipolysis (Fig. 5).

Metabolomic profiling studies indicated that FAAH^−/−^ deletion resulted in overall depletion of hepatic acylcarnitine pools (Fig. 3a). While this reflects increased fatty acid oxidation, lipogenesis chronically is not increased. TAG accumulation in FAAH^−/−^ liver is a result of increased re-esterification of plasma free fatty acids coming from the unsuppressed adipose lipolysis in the fasted state, and increased levels of lipin 1 and DGAT 1 in the fed state.

More specifically, the liver acyl-carnitine profiling detailed a decrease in fasted acetyl carnitine, suggesting a decrease of acetyl-CoA, as acetyl-CoA is buffered via conversion to acetyl carnitine [32]. This is supported by acetyl-CoA measurements, which show an increase in acetyl-CoA from the fasted to re-fed state for FAAH^−/−^ mice, as opposed to the wild-type mice (Fig. 3b). Decreased fasted and increased fed citrate levels in FAAH^−/−^ versus wild-type livers support the increased fed production of acetyl-CoA.

Feedback modulation of the metabolic network is heavily influenced by acetylation, which serves, in part, as a monitor of acetyl-CoA levels. Approximately 2000 acetylated proteins have been previously identified in mammalian cells [33] and prokaryotic cells [34] and metabolic enzymes are highly represented. It has been postulated, that acetylation serves to coordinate flux in the central metabolism network, as nearly all enzymes involved in glycolysis, gluconeogenesis, the TCA cycle, FAO, the urea cycle, glycogen metabolism, oxidative phosphorylation, and amino acid metabolism are acetylated [22], [35], [36]. Therefore, we investigated the impact of FAAH deficiency on the hepatic metabolic acetylome. We compared mitochondrial and cytosolic protein acetylation changes, to metabolite profiling changes seen in glycolytic, pentose and TCA cycle, as well as how it relates to the SIPHEN assessment of the rate of hepatic biosynthetic processes, such as HGP, lipogenesis and cholesterol synthesis.

FAAH^−/−^ livers had decreased ACAT1 acetylation in the fasted state. A decrease in 3-ketoacyl-CoA thiolase/trifunctional protein acetylation was observed in fed FAAH^−/−^ livers (Fig. 8). Both of these enzymes affect the balance between acetyl-CoA and acetoacetyl-CoA levels, supported by the changes seen in acetyl-CoA levels (Fig. 3b).

Hydroxymethylglutaryl-coenzyme A (HMG-CoA), the precursor for cholesterol synthesis is formed from the condensation of acetyl-CoA and acetoacetyl CoA. The decrease in cholesterol synthesis seen in FAAH^−/−^ mice may be reflective of a disturbance in acetyl-CoA levels arising from the imbalance in ACAT1 and 3-ketoacyl-CoA thiolase acetylation states. Hepatic metabolite profiling illustrates the functional importance of dysregulated lysine acetylation on flux through the TCA and glycolytic pathways. It also suggests linkages to dysregulation in hepatic FAO and cholesterol synthesis.

Acetylation of mitochondrial MDH2 can increase its activity [22], [33]. Acetylation of aldolase B can decrease its activity [37], [38]. Altered MDH2 activity has potential effects on hepatic energy homeostasis for (dys)regulation of the malate aspartate shuttle, and the TCA cycle [39]. The changes in acetylation for mitochondrial MDH2, which was hypoacetylated in fasted FAAH^−/−^ livers, and hyperacetylated in fed FAAH^−/−^ livers supports the metabolite profiling indicating an impairment in the malate/aspartate shuttle. While DHAP and glycerol-3-P levels were decreased in the fasted state of the FAAH^−/−^ mice, they were preserved in the fed state, consistent with a compensating contribution from a decrease in fed aldolase B acetylation in FAAH^−/−^ mice.

In the fed state, under normal conditions, HGP is suppressed by insulin, and carbohydrate is used as a predominant energy source. We hypothesize that the hepatic insulin resistance (unsuppressed glucose production and impaired fuel switching) seen in the pre-diabetic FAAH^−/−^ mouse can be attributed, in part, to its dysregulated lysine acetylation. In our study, acetylation status of different proteins differed greatly, as for example, fructose bisphosphate aldolase B (gluconeogenic) and mitochondrial 3 keto-acyl thiolase (fatty acid oxidizing) enzymes were hypoacetylated, and ATP synthase coupling factor 6 and malate dehydrogenase (mitochondrial) were hyperacetylated, in the fed FAAH^−/−^ versus wild-type livers (Figure 8 and Table S3). Acetylation changes associated with fasted to fed transitions in wild-type versus FAAH^−/−^ mice differed greatly. The observation of these mixed acetylation changes can be attributed to existence of different cellular pools and sub-pools of liver mitochondrial acetyl-CoA and N-acetyl transferases [40] with hepatic extra-mitochondrial acetyl-CoA accounting for less than 5% of the total pool. Further, Zhang et al [41] have shown that sub pools of acetyl-CoA, derived from different sources, can stream past each other. Their work implies that competition between drugs being acetylated alters the labeling of individual acetyl-CoA sub-pools, and it may be competition between proteins being acetylated also affects acetylation, in addition to acetyl-CoA compartmentation. While acetylation of MDH2 and ATP synthase subunit d changed in opposite directions, acetylation changes for phosphoglycerate mutase 2 and aspartate aminotransferase observed in wild-type mice were not evident for FAAH^−/−^ mice. These changes may be reflected in the differences seen for malate, aspartate and glycolytic intermediates. The differences in acetylation for ACAT1 seen in the fasted to fed transition for FAAH^−/−^ mice, not seen for wild-type, may be reflected in the differences seen in the rate of cholesterol synthesis. The decreased acetylation of alcohol dehydrogenase in fed FAAH^−/−^ mice, suggests that elevated hepatic endocannabinoids could alter alcohol metabolism, depending on dietary state.

In conclusion, our study suggests that FAAH^−/−^ mice are a model of the pre-diabetic state, having adipose, skeletal and hepatic insulin resistances. While fuel switching in skeletal muscle is preserved, HGP is unsuppressed with impaired glucose tolerance. Collectively, we propose that dysregulation of lysine acetylation occurs with impaired FAA metabolism and could support the liver's role in fostering the pre-diabetic state seen with impaired FAA hydrolysis. FAAH deficiency also promotes insulin resistance and liver steatogenesis, two key steps in the pathogenesis of non-alcoholic fatty liver disease, and CB_1_ antagonists have been proposed in the management of NASH [42]. Elevated liver endocannaboids have been implicated also in the pathogenesis of alcoholic liver disease [43], [44]. Our FAAH^−/−^ hepatic acetylome findings share similarities with the global ALD acetylome found by Shepard and Tuma [45], which also found acetylation dysregulation of 3-ketoacyl-CoA thiolase, acetyl-CoA acetyltransferase, fructose-bisphosphate aldolase B, superoxide dismutase and a change in ATP synthase, but in subunit b. Also inhibitors of FAAH are currently in development for the treatment of pain and inflammation [46], making the characterization of the effects of FAAH inhibition of further clinical interest. Further work will establish the linkage between FAAs and disorders of lysine acetylation, in the development of obesity, NASH, ALD and Type II DM.

## Materials and Methods

### Ethics Statement

All animal studies were performed under approved institutional protocols and according to guidelines established in the Guide for the Care and Use of Laboratory Animals. The animal protocols were in accordance with IACUC (Institutional Animal Care and Use Committee) - Albert Einstein College of Medicine of Yeshiva University approval # 20090308 and State University of New York, Stony Brook approval # 1346.

#### Animals

FAAH^−/−^ mice backcrossed at least 6 generations onto a C57BL/6 background were a generous gift from Dr. Benjamin F Cravatt (Scripps Research Institute, La Jolla, CA). Consistent with our previously published work [47], [48], and that of others [49], age matched C57BL/6 mice were used as wild-type controls. All animals were males, 4–5 months of age, and fed standard laboratory chow diet (4.5% fat, 20% protein and 54.8% carbohydrate by weight) (PICOLab Rodent Diet 20; 5053). Animals were maintained under 12 h light/dark conditions (0700 h/1900 h) for all studies.

Animals from calorimetry experiments (n = 8) were used for body composition analyses and sacrificed at the end of the D_2_O lipogenesis experiments. Five animals each from stable isotope glucose tolerance test (n = 10) were finally sacrificed with Alzet osmotic minipump SIPHEN studies. Livers from the 18 h fast n = 4/5 h re-fed experiment n = 4 without tracer were used for hepatic metabolite profiling, immunoblot, glycogen, lipid TLC, acetyl-CoA, acetyl carnitine and acetylome analyses.

### Body Composition

Body composition was determined by low resolution nuclear magnetic resonance (NMR) using a benchtop pulsed NMR (7 T) system (Minispec Model mq7.5 (7.5 mHz) manufactured and tested by Bruker Instruments) at Vanderbilt mouse phenotyping centre. Percent lean mass and fat mass were calculated as a proportion of body weight. FAAH^−/−^ mice and age matched wild-type C57BL/6 mice were examined.

### Indirect Calorimetry

Measurements of oxygen consumption (VO_2_) and respiratory quotient (RER) were performed using an Oxymax indirect calorimetry system (Columbus Instruments, Columbus, OH). Mice (n = 8/genotype) were housed individually in the chamber for 48 h with lights on from 0700 to 1900 h at an ambient temperature of 22–24°C. Food was available ad libitum during the dark cycle (feeding phase 1900–0700 h) and the light cycle (fasting phase 0700–1900 h). Gas exchange measurements were made under Oxymax system settings as follows: air flow, 0.6 l/min; sample flow, 0.5 l/min; settling time, 6 min; and measuring time, 3 min. Ambulatory activity was determined simultaneously using an Opto-Varimetrix-3 sensor system. Consecutive adjacent infrared beam breaks in either the *x*- or *y*-axes were scored as an activity count.

### De novo lipogenesis, cholesterol synthesis and protein synthesis

Lipogenesis, cholesterol and protein synthesis were measured using deuterated water [^2^H_2_O], tracing the enrichment of deuterium in palmitate, cholesterol and alanine respectively. Briefly, mice received an intraperitoneal injection of deuterated water (^2^H_2_O, at a concentration of 4% lean body mass) containing 0.9% sodium chloride. Mice received 4% ^2^H_2_O as drinking water and were left on 4% ^2^H_2_O for 10 days with free access to chow. Mice were sacrificed at the end of tenth day following a 4 h fast (0700–1100 h). Blood was collected from the retro-orbital sinus, liver and skeletal muscle (quadriceps) were removed and snap frozen in liquid nitrogen. Plasma was used for measuring body deuterium enrichment.

Palmitate and cholesterol were analyzed as their trimethylsilyl derivatives using gas chromatography (GC) -electron impact ionization mass spectrometry (MS) as previously described [50], [51], [52], [53].

For protein synthesis studies, alanine enrichment was measured and protein synthesis rate was calculated as previously described [54], [55].

### Stable Isotope Flux Phenotyping (SIPHEN)

SIPHEN studies included assessment of hepatic glucose production ([U-^13^C_6_]-glucose), glycerol production ([2-^13^C_2_]-glycerol) using Alzet mini-osmotic pumps and a (SipGTT) stable isotope labeled glucose tolerance test ([6,6-2H2]-glucose). All stable isotopes were purchased from Cambridge Isotope Laboratories (Andover, MA) and were pyrogen tested. Blood samples were analyzed on a GC/MS. Details can be found in our previous studies [30], [31], [56], [57], [58], [59], [60].

### Serum Analysis

Plasma glucose levels were determined by COBAS MIRA analyzer (Roche, Montclair, NJ) using the Glucose UV Reagent (catalog no. 80017, Raichem, San Diego, CA). Plasma insulin was determined using an ultra-sensitive rat/mouse Insulin ELISA Kit with intra-assay precision CV≤10.0% and 0.1–6.4 ng/ml limit detection (Crystal Chem. Inc., Cat# 90060). NEFA (WAKO HR Series NEFA-HR (2) Kit, cat # 997-76491), cholesterol (WAKO Cholesterol E kit cat # 439-17501) and TG levels (Triglycerides Reagent kit from Thermo scientific cat # TR22203/2750-500) were used to determine plasma NEFA, cholesterol and TG levels respectively.

### Acetyl-CoA and Acylcarnitine measurements

Hepatic acetyl-CoA in the fasted and re-fed liver samples was measured according to [61]. Briefly, frozen pulverized liver samples were homogenized in phosphate buffered saline (pH 7.2) and were acidified to 0.1% formic acid. After centrifugation to get rid of cell debris and proteins, the supernatent was collected and pH was adjusted to 7.5 with 2–3 drops 50% ammonium hydroxide and taken for LC/MS analysis.

Hepatic acylcarnitines in fasted and re-fed liver samples were determined using LC tandem MS as described previously [62] with slight modifications for sample preparation. Briefly pulverized liver samples were extracted in 1∶1∶3 (water∶methanol∶acetonitrile) acidified to 0.1% formic acid. Supernatent from spun down samples were lypophilized, redissolved in 0.1% formic acid, filtered and used for LC/MS analysis. LC/MS analysis was performed in a Waters Acquity UPLC system attached to a Xevo Triple Quadrupole mass spectrometer equipped with an electrospray ionization source (ESI) (Waters Corp., Milford, MA).

### Label-Free Quantitative Assessment of the Fasted/Re-fed Hepatic Acetylome

Mice (n = 4 in each group) were sacrificed following either an 18 h fast (2030 h–1430 h) or a 13 h fast (2030 h–0930 h) followed by 5 h re-feeding (0930–1430 h). Proteins were extracted from harvested livers that were snap frozen and stored in liquid nitrogen. Around 30 mg of pooled protein/group (7.5 mg from each mouse) was used for hepatic acetylome analysis. Identification and quantification of acetylated proteins was done using immunoprecipitation followed by liquid chromatography (LC), MS/MS experiments on a home-built high accuracy mass spectrometer (Velos FT-ICR) (Weisbrod et al, submitted RCMS). A detailed description for methodology and calculations can be found in our recent publication [22].

### Hepatic Metabolite Profiling

Freeze clamped liver samples from fasted/re-fed experiments were extracted in ice-cold methanol/water (1∶1) spiked with internal standards (U-^13^C_4_ succinate and U-^13^C_6_ citric acid, (150 n moles/gm liver). Lipids were removed by shaking with an equal volume of chloroform. The aqueous phase was lyophilized, derivatized [63] and GC/MS analysis performed using an Agilent 7890a GC with a Gerstel automatic liner exchange cooled injection system CIS 4 PTV injector. Both split and large volume injections (LVI) of the sample were made. GC/MS conditions and metabolite identification was done as described [64] using NIST 11 and Fiehn mass spectral libraries.

### Lipid Content Analysis

Lipid content analysis (DAGs and TAGs) was performed using thin layer chromatography as described [65]. Briefly, frozen tissues (quadriceps, liver) isolated from animals sacrificed at the end of 18 h fast, 5 h refeeding experiments were weighed, and Bligh-Dryer extraction for tissue lipid was performed. The extracted lipid was evaporated under N_2_ in glass tubes to dryness and re-dissolved in chloroform∶methanol∶acetic acid∶water (25∶15∶4∶2) normalized by the weight of the tissue taken to 1 mg/ml solvent. 10 µl (liver) and 50 µl (muscle) of the each sample was spotted to their respective lane on a silica gel plate (Whatman Partisil K6). TLC was then performed using the conventional three solvent system. An aliquot of the lipid extract was dried and resuspended in 5% NP-40 solution and quantified for triglycerides using Triglycerides Reagent kit from Thermo scientific (cat # TR22203/2750-500).

### Determination of Glycogen Content

Frozen tissue (quadriceps, liver) isolated from animals sacrificed at the end of 18 h fast, 5 h re-feeding experiments were weighed homogenized in ice-cold 6% perchloric acid. The resultant lysate was spun at 2000 rpm for 20 minutes and supernatant containing glycogen was saved. Glycogen was then precipitated with 5 volumes of ice-cold 100% ethanol and left on ice over-night at 4°C to ensure complete precipitation. The precipitate was collected by spinning at 5000 rpm/20 minutes. Ethanol precipitation was repeated one more time to remove any free glucose. The resulting clean glycogen, was dissolved in 0.1 M sodium acetate buffer (pH 4.5), and incubated in 50 µl of amyloglucosidase overnight at 37°C. Glycogen hydrolyzed to glucose by the overnight enzyme reaction was quantitated for glucose colorimetrically using Quantichrom Glucose assay reagent (cat # DIGL-200).

### Immunoblot Analysis

Frozen liver was homogenized in ice-cold lysis buffer (50 mM Tris, pH 7.5, 150 mM NaCl, 1% Triton X-100, 1 mM EDTA, 1 mM phenylmethylsulfonyl fluoride, 0.25% sodium deoxycholate, 1 mM NaF, 1 mM Na_3_VO_4_, and 2 mM Na_4_P_2_O_7_) containing a protease inhibitor mixture (Roche Diagnostics) and also deacetylase inhibitors for anti-lysine western. The resultant lysates were centrifuged at 16,000×*g* for 60 min at 4°C, and protein concentrations were quantified using the BCA (bicinchoninic acid) protein assays (Pierce, Inc.). The protein samples (30 µg) were separated on an 8% gradient SDS-PAGE gel and transferred to PVDF membranes using a semidry electroblotter (BioRad). Membranes were immunoblotted (Antibodies were purchased from Cell Signalling, Santa Cruz Biotech Inc. and Immunechem Pharmaceuticals Inc.) and signals were visualized and quantified using the infrared Odyssey Western Blotting System (Li-Cor, Lincoln, NE).

### Statistical Analyses

Data are expressed as the mean ± SEM. Analyses for the significance of differences were performed using the Student's t-test and Anova using GraphPad Prism version 5.00 for Windows, GraphPad Software, San Diego California USA, www.graphpad.com.

## Supporting Information

Figure S1 Immunoblot analysis for fasted/fed expression of key metabolic proteins. Immunoblot analysis for fasted/fed expression of key metabolic proteins FAS, ACL, G6PDH, Rheb, PF2K, GCK, AMPK, ACC involved in hepatic fuel switching showing no differences in their levels.
(TIF)

Figure S2 Distribution of log_10_ values for all the quantified acetylated liver peptides. a) fasted FAAH^−/−^ vs. wild-type and b) re-fed FAAH^−/−^ vs. wild-type. The mean and standard deviation values were calculated under the assumption that the data follow normal distribution. The top-5% dramatically changed peptides were determined by those outside mean ± two-standard deviations.
(TIF)

Figure S3 FAAH deficiency increases fed levels of hepatic and skeletal muscle triglycerides. Quantification of fed hepatic and muscle triglycerides in FAAH^−/−^ vs. wild-type mice. n = 4, data are mean ± SEM, **p<0.01 by Student's t-test.
(TIF)

Figure S4 FAAH deficiency alters fasted/fed hepatic protein lysine acetylation. a. Global representation of hepatic protein lysine acetylation by immunopreciptiation and immunoblot analysis with anti-acetylated lysine antibodies to detect acetylated proteins in 18 h fasted and 5 h re-fed FAAH^−/−^ vs. wild-type mice (n = 4). b. Western analysis on the anti lysine IP for two different acetylation target proteins (alcohol and malate dehydrogenase) done as an example to show differential fasting to re-feeding regulation by acetylation. The flow through from the anti-lysine immunoprecipitation was used for probing β actin as a loading control.
(TIF)

Table S1 Summary containing list of total identified acetylated proteins and acetylated peptides from the hepatic acetylome.
(XLSX)

Table S2 List of largely altered acetylated peptides, their sequences, protein origins with their index values. In total, 49 acetylated peptides from 12 different proteins that were altered are presented. Validated peptides with a common sequence but modifications at different sites are treated as different entries in the table. An index number was given to each entry. Multiple acetylated peptides from the same protein were grouped in the list, so they have neighboring index numbers. @ represents acetylated lysine residues and $ represents, oxidized methionine residues.
(DOC)

Table S3 Top 5% dramatically changed acetylated peptides (>4-fold change). The acetylated peptides and corresponding proteins that showed more than a four-fold change between 18 h fast/5 h fed FAAH^−/−^ and wild-type mice were compared and summarized in this table. @, $ indicate the acetylated and oxidized sites, respectively.
(DOC)
