# Supplementary figures and images for: Peripheral Effects of FAAH Deficiency on Fuel and Energy Homeostasis: Role of Dysregulated Lysine Acetylation
Source: PLoS One. 2012 Mar 19;7(3):e33717. doi: 10.1371/journal.pone.0033717 (PMC3307749; doi:10.1371/journal.pone.0033717)

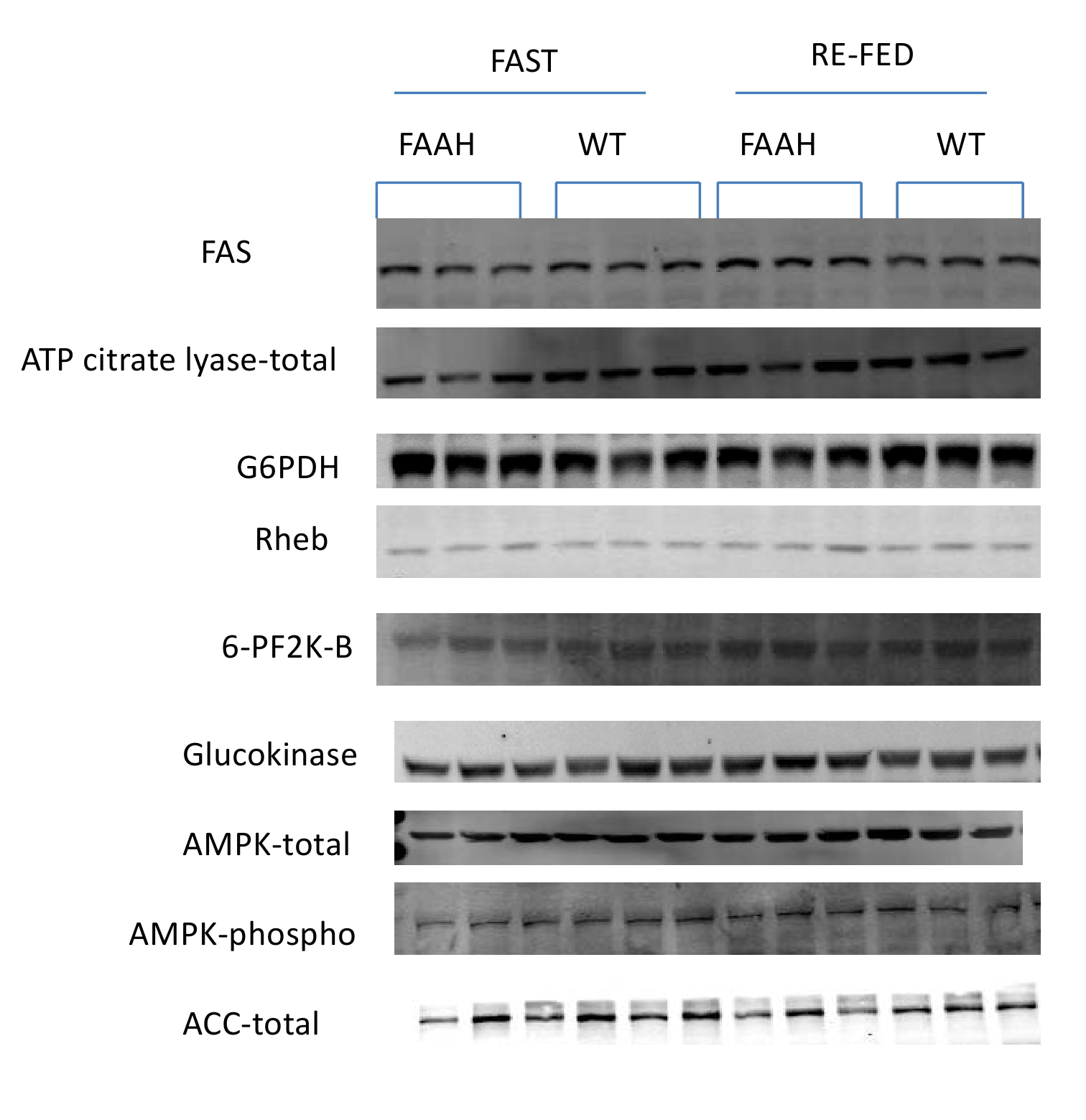

Supplement: Figure S1 — Immunoblot analysis for fasted/fed expression of key metabolic proteins. Immunoblot analysis for fasted/fed expression of key metabolic proteins FAS, ACL, G6PDH, Rheb, PF2K, GCK, AMPK, ACC involved in hepatic fuel switching showing no differences in their levels. (TIF) [file pone.0033717.s001.tif]

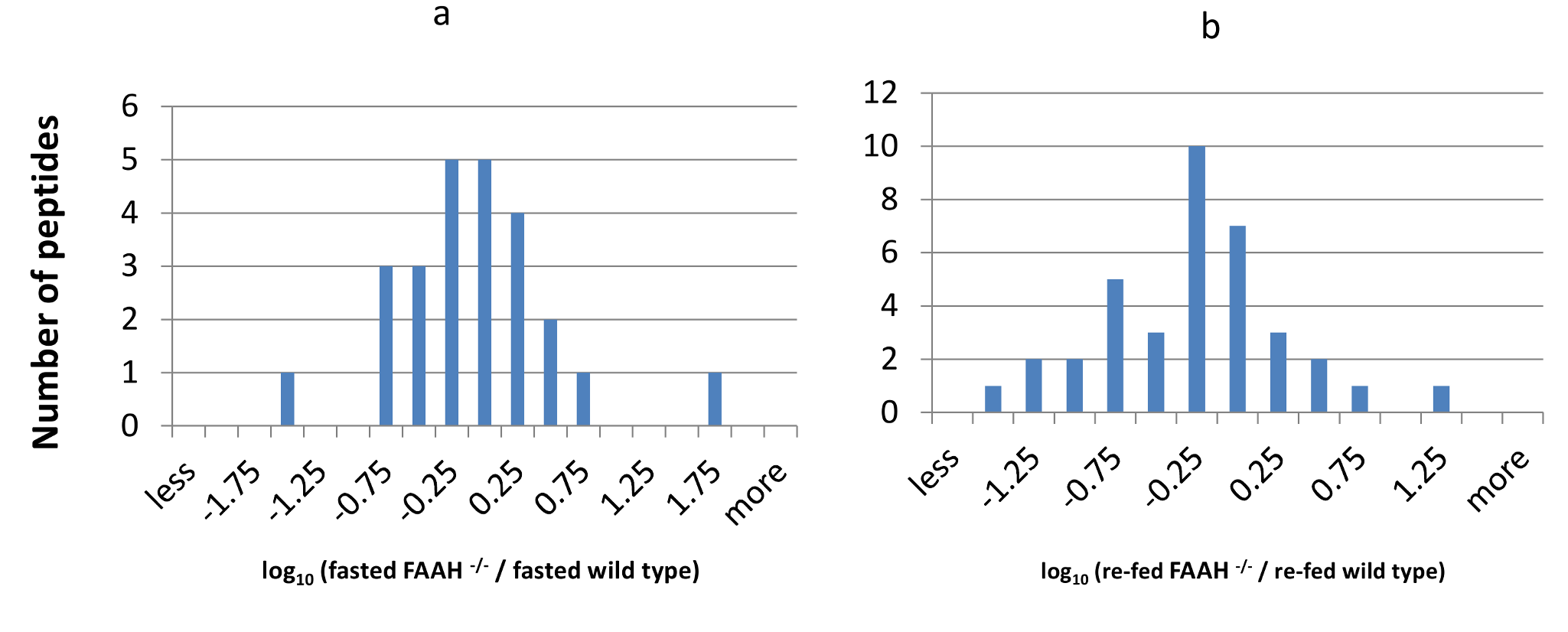

Supplement: Figure S2 — Distribution of log10 values for all the quantified acetylated liver peptides. a) fasted FAAH−/− vs. wild-type and b) re-fed FAAH−/− vs. wild-type. The mean and standard deviation values were calculated under the assumption that the data follow normal distribution. The top-5% dramatically changed peptides were determined by those outside mean ± two-standard deviations. (TIF) [file pone.0033717.s002.tif]

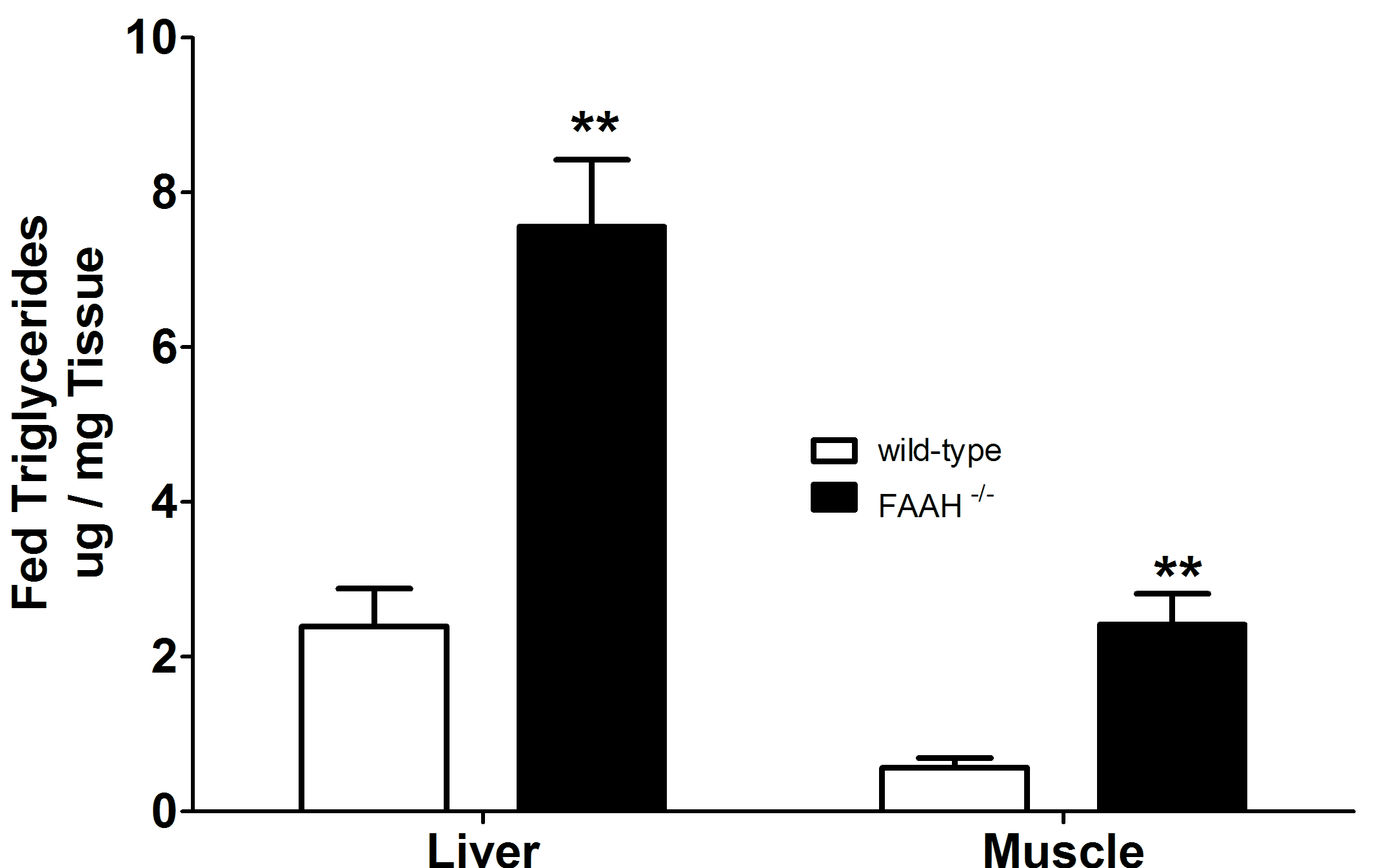

Supplement: Figure S3 — FAAH deficiency increases fed levels of hepatic and skeletal muscle triglycerides. Quantification of fed hepatic and muscle triglycerides in FAAH−/− vs. wild-type mice. n = 4, data are mean ± SEM, **p<0.01 by Student's t-test. (TIF) [file pone.0033717.s003.tif]

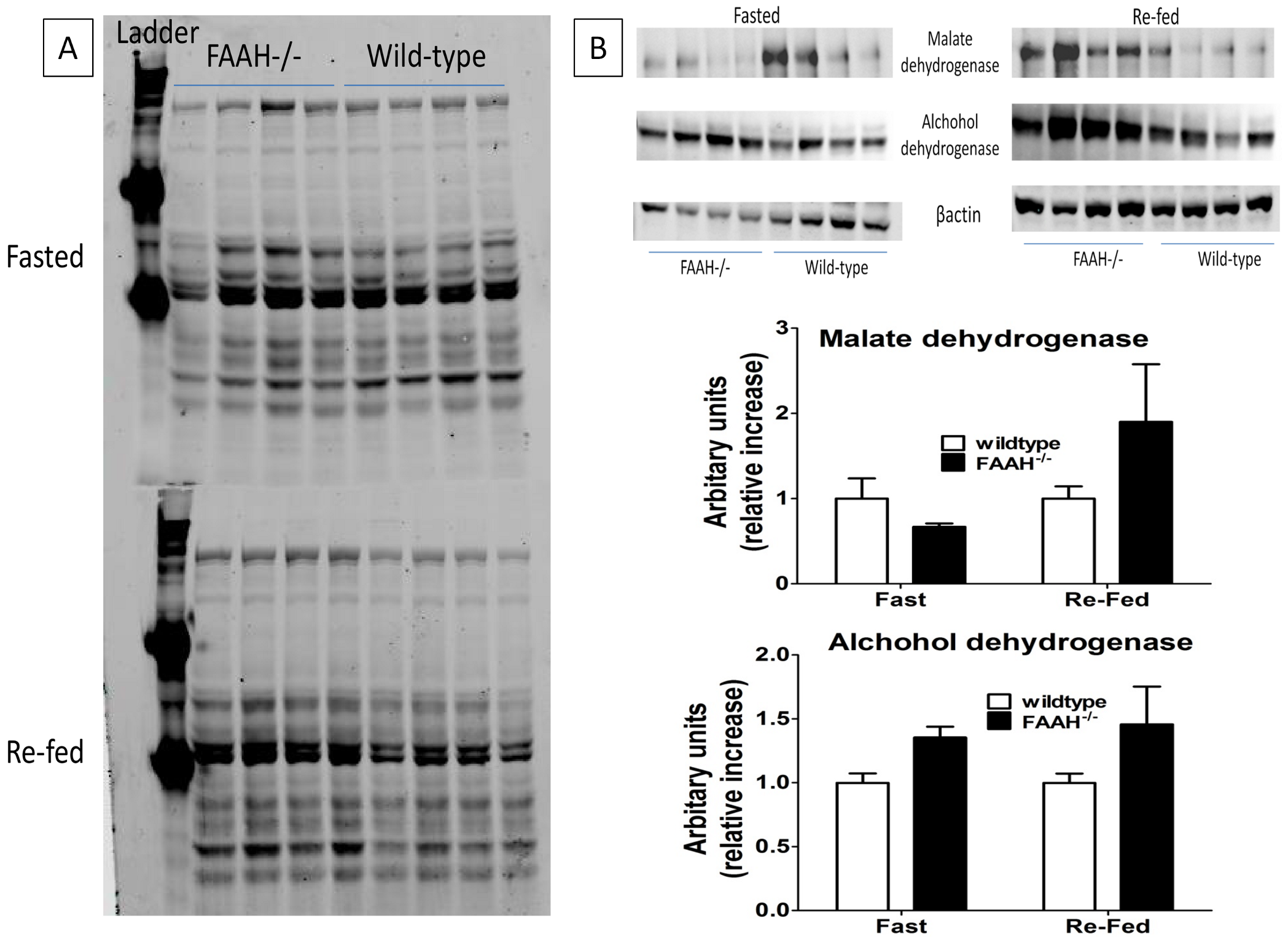

Supplement: Figure S4 — FAAH deficiency alters fasted/fed hepatic protein lysine acetylation. a. Global representation of hepatic protein lysine acetylation by immunopreciptiation and immunoblot analysis with anti-acetylated lysine antibodies to detect acetylated proteins in 18 h fasted and 5 h re-fed FAAH−/− vs. wild-type mice (n = 4). b. Western analysis on the anti lysine IP for two different acetylation target proteins (alcohol and malate dehydrogenase) done as an example to show differential fasting to re-feeding regulation by acetylation. The flow through from the anti-lysine immunoprecipitation was used for probing β actin as a loading control. (TIF) [file pone.0033717.s004.tif]
