# Supplementary material for: Peripheral Effects of FAAH Deficiency on Fuel and Energy Homeostasis: Role of Dysregulated Lysine Acetylation
Source: PLoS One. 2012 Mar 19;7(3):e33717. doi: 10.1371/journal.pone.0033717 (PMC3307749; doi:10.1371/journal.pone.0033717)
Supplement: Table S2 — List of largely altered acetylated peptides, their sequences, protein origins with their index values. In total, 49 acetylated peptides from 12 different proteins that were altered are presented. Validated peptides with a common sequence but modifications at different sites are treated as different entries in the table. An index number was given to each entry. Multiple acetylated peptides from the same protein were grouped in the list, so they have neighboring index numbers. @ represents acetylated lysine residues and $ represents, oxidized methionine residues. (DOC) [file pone.0033717.s006.doc]

Table S2. List of largely altered acetylated peptides, their sequences, protein origins with their index values.

| **Index** | **Protein accession number** | **Protein name** | **Peptide sequence** | **Theoretical mass** | **Except score** |
| --- | --- | --- | --- | --- | --- |
| 1 | IPI00109109 | Superoxide dismutase [Mn], mitochondrial precursor | DFGSFEK@FK | 1145.5393 | 0.08 |
| 2 | IPI00109109 | Superoxide dismutase [Mn], mitochondrial precursor | DFGSFEK@FKEK | 1402.6769 | 0.0096 |
| 3 | IPI00109109 | Superoxide dismutase [Mn], mitochondrial precursor | HHAAYVNNLNATEEK@YHEALAK | 2564.2407 | 0.00016 |
| 4 | IPI00117312 | Aspartate aminotransferase, mitochondrial precursor | DDNGK@PYVLPSVR | 1500.7572 | 0.079 |
| 5 | IPI00117312 | Aspartate aminotransferase, mitochondrial precursor | KMNLGVGAYRDDNGK@PYVLPSVR | 2590.3326 | 0.038 |
| 6 | IPI00117312 | Aspartate aminotransferase, mitochondrial precursor | KM$NLGVGAYRDDNGK@PYVLPSVR | 2606.3275 | 0.07 |
| 7 | IPI00117312 | Aspartate aminotransferase, mitochondrial precursor | MNLGVGAYRDDNGK@PYVLPSVR | 2462.2376 | 0.063 |
| 8 | IPI00117312 | Aspartate aminotransferase, mitochondrial precursor | M$NLGVGAYRDDNGK@PYVLPSVR | 2478.2325 | 0.11 |
| 9 | IPI00117312 | Aspartate aminotransferase, mitochondrial precursor | TQLVSNLK@K | 1071.6288 | 0.1 |
| 10 | IPI00119006 | T-lymphoma invasion and metastasis-inducing protein 1 | VPK@VLVK | 823.5531 | 0.041 |
| 11 | IPI00125460 | ATP synthase coupling factor 6, mitochondrial precursor | FDDPK@FEVIDKPQS | 1705.8199 | 0.017 |
| 12 | IPI00125460 | ATP synthase coupling factor 6, mitochondrial precursor | FDDPKFEVIDK@PQS | 1705.8199 | 0.025 |
| 13 | IPI00125460 | ATP synthase coupling factor 6, mitochondrial precursor | GEM$DTFPTFK@FDDPK | 1831.7975 | 0.047 |
| 14 | IPI00125460 | ATP synthase coupling factor 6, mitochondrial precursor | GEMDTFPTFK@FDDPKFEVIDKPQS | 2859.3313 | 0.091 |
| 15 | IPI00125460 | ATP synthase coupling factor 6, mitochondrial precursor | GEM$DTFPTFK@FDDPKFEVIDKPQS | 2875.3262 | 0.07 |
| 16 | IPI00125460 | ATP synthase coupling factor 6, mitochondrial precursor | GEM$DTFPTFKFDDPK@FEVIDKPQS | 2875.3262 | 0.11 |
| 17 | IPI00125460 | ATP synthase coupling factor 6, mitochondrial precursor | LFVDK@IR | 931.5491 | 0.02 |
| 18 | IPI00125460 | ATP synthase coupling factor 6, mitochondrial precursor | QMYGKGEM$DTFPTFKFDDPK@FEVIDKPQS | 3482.6051 | 0.071 |
| 19 | IPI00127206 | Fructose-bisphosphate aldolase B | DGVDFGK@WR | 1120.5302 | 0.072 |
| 20 | IPI00154054 | Acetyl-CoA acetyltransferase, mitochondrial precursor | AGIPK@EEVKEVYM$GNVIQGGEGQAPTR | 2914.4494 | 0.054 |
| 21 | IPI00154054 | Acetyl-CoA acetyltransferase, mitochondrial precursor | AGIPKEEVK@EVYM$GNVIQGGEGQAPTR | 2914.4494 | 0.1 |
| 22 | IPI00154054 | Acetyl-CoA acetyltransferase, mitochondrial precursor | EAWDAGK@FASEITPITISVK | 2204.1365 | 0.0017 |
| 23 | IPI00154054 | Acetyl-CoA acetyltransferase, mitochondrial precursor | GATPYGGVK@LEDLIVK | 1700.9349 | 0.011 |
| 24 | IPI00154054 | Acetyl-CoA acetyltransferase, mitochondrial precursor | GATPYGGVK@LEDLIVKDGLTDVYNK | 2706.4116 | 0.0032 |
| 25 | IPI00154054 | Acetyl-CoA acetyltransferase, mitochondrial precursor | SK@EAWDAGKFASEITPITISVK | 2419.2635 | 0.04 |
| 26 | IPI00154054 | Acetyl-CoA acetyltransferase, mitochondrial precursor | SKEAWDAGK@FASEITPITISVK | 2419.2635 | 0.00051 |
| 27 | IPI00221769 | GTP:AMP phosphotransferase mitochondrial | HFELK@HLSSGDLLR | 1692.8947 | 0.0013 |
| 28 | IPI00221769 | GTP:AMP phosphotransferase mitochondrial | ITKHFELK@HLSSGDLLR | 2035.1214 | 0.00056 |
| 29 | IPI00226430 | 3-ketoacyl-CoA thiolase, mitochondrial | FGLDLK@LEDTLWAGLTDQHVK | 2440.2638 | 0.03 |
| 30 | IPI00226430 | 3-ketoacyl-CoA thiolase, mitochondrial | FGTK@FGLDLK | 1166.6336 | 0.097 |
| 31 | IPI00226430 | 3-ketoacyl-CoA thiolase, mitochondrial | K@HNFTPLAR | 1124.6091 | 0.095 |
| 32 | IPI00226430 | 3-ketoacyl-CoA thiolase, mitochondrial | LPMGMTAENLAAK@YNISR | 2021.0074 | 0.01 |
| 33 | IPI00226430 | 3-ketoacyl-CoA thiolase, mitochondrial | LPM$GMTAENLAAK@YNISR | 2037.0023 | 0.0021 |
| 34 | IPI00226430 | 3-ketoacyl-CoA thiolase, mitochondrial | LPMGM$TAENLAAK@YNISR | 2037.0023 | 0.037 |
| 35 | IPI00226430 | 3-ketoacyl-CoA thiolase, mitochondrial | LPM$GM$TAENLAAK@YNISR | 2052.9972 | 0.093 |
| 36 | IPI00226430 | 3-ketoacyl-CoA thiolase, mitochondrial | QTMQVDEHARPQTTLEQLQK@LPSVFKK | 3221.6867 | 0.047 |
| 37 | IPI00230507 | ATP synthase D chain, mitochondrial | ANVAKPGLVDDFEK@K | 1671.8832 | 0.00036 |
| 38 | IPI00230507 | ATP synthase D chain, mitochondrial | ANVAKPGLVDDFEK@KYNALK | 2261.2055 | 0.0026 |
| 39 | IPI00230507 | ATP synthase D chain, mitochondrial | IPVPEDK@YTALVDQEEKEDVK | 2486.2428 | 0.1 |
| 40 | IPI00230507 | ATP synthase D chain, mitochondrial | IQEYEK@QLEK | 1348.6874 | 0.069 |
| 41 | IPI00230507 | ATP synthase D chain, mitochondrial | K@YPYWPHQPIENL | 1725.8515 | 0.018 |
| 42 | IPI00230706 | Phosphoglycerate mutase 2 | HYGGLTGLNK@AETAAK | 1671.858 | 0.0068 |
| 43 | IPI00323592 | Malate dehydrogenase, mitochondrial precursor | GEDFVK@NM$K | 1124.5172 | 0.055 |
| 44 | IPI00323592 | Malate dehydrogenase, mitochondrial precursor | GLEK@NLGIGK | 1069.6131 | 0.021 |
| 45 | IPI00323592 | Malate dehydrogenase, mitochondrial precursor | ITPFEEK@MIAEAIPELK | 2000.054 | 0.078 |
| 46 | IPI00323592 | Malate dehydrogenase, mitochondrial precursor | ITPFEEK@M$IAEAIPELK | 2016.0489 | 0.041 |
| 47 | IPI00323592 | Malate dehydrogenase, mitochondrial precursor | KGEDFVK@NMK | 1236.6172 | 0.0055 |
| 48 | IPI00323592 | Malate dehydrogenase, mitochondrial precursor | KGEDFVK@NM$K | 1252.6122 | 0.017 |
| 49 | IPI00466128 | Alcohol dehydrogenase | GDNPFPK@NADGTVR | 1528.727 | 0.031 |
