# Supplementary material for: Peripheral Effects of FAAH Deficiency on Fuel and Energy Homeostasis: Role of Dysregulated Lysine Acetylation
Source: PLoS One. 2012 Mar 19;7(3):e33717. doi: 10.1371/journal.pone.0033717 (PMC3307749; doi:10.1371/journal.pone.0033717)
Supplement: Table S3 — Top 5% dramatically changed acetylated peptides (>4-fold change). The acetylated peptides and corresponding proteins that showed more than a four-fold change between 18 h fast/5 h fed FAAH−/− and wild-type mice were compared and summarized in this table. @, $ indicate the acetylated and oxidized sites, respectively. (DOC) [file pone.0033717.s007.doc]

Table S3. Top 5% dramatically changed acetylated peptides (> 4-fold change).

| **log_10_ (fasted FAAH ^-/-^ / fasted wild type)** | | | | |
| --- | --- | --- | --- | --- |
| **Protein accession number** | **Peptide sequence** | **log10 Ratio** | **Protein name** | **Index** |
| IPI00230507 | K.K@YPYWPHQPIENL.- | 1.99 | ATP synthase subunit d, mitochondrial | 41 |
| IPI00154054 | R.GATPYGGVK@LEDLIVK.D | -0.75 | Acetyl-CoA acetyltransferase, mitochondrial | 23 |
| IPI00323592 | K.ITPFEEK@M$IAEAIPELK.A | -1.41 | Malate dehydrogenase, mitochondrial | 46 |
|  |  |  |  |  |
| **log_10_ (fasted FAAH ^-/-^ / re-fed FAAH ^-/-^)** | | | | |
| **Protein accession number** | **Peptide sequence** | **log10 Ratio** | **Protein name** | **Index** |
| IPI00323592 | K.ITPFEEK@M$IAEAIPELK.A | -1.21 | Malate dehydrogenase, mitochondrial | 46 |
| IPI00221769 | K.HFELK@HLSSGDLLR.Q | -0.62 | GTP:AMP phosphotransferase, mitochondrial | 27 |
| IPI00109109 | R.DFGSFEK@FKEK.L | -0.75 | Superoxide dismutase [Mn], mitochondrial | 2 |
|  |  |  |  |  |
| **log_10_ (wild type fasted / wild type re-fed)** | | | | |
| **Protein accession number** | **Peptide sequence** | **log10 Ratio** | **Protein name** | **Index** |
| IPI00125460 | K.GEM$DTFPTFK@FDDPKFEVIDKPQS.- | 1.43 | ATP synthase-coupling factor 6, mitochondrial | 15 |
| IPI00323592 | K.ITPFEEK@M$IAEAIPELK.A | 1.28 | Malate dehydrogenase, mitochondrial | 46 |
| IPI00119006 | K.VPK@VLVK.A | 1.29 | T-lymphoma invasion and metastasis-inducing protein 1 | 10 |
| IPI00230507 | K.K@YPYWPHQPIENL.- | -2.28 | ATP synthase subunit d, mitochondrial | 41 |
| IPI00117312 | K.KMNLGVGAYRDDNGK@PYVLPSVR.K | -2.17 | Aspartate aminotransferase, mitochondrial | 5 |
| IPI00230706 | R.HYGGLTGLNK@AETAAK.H | -1.46 | Phosphoglycerate mutase 2 | 42 |
|  |  |  |  |  |
| **log_10_ (re-fed FAAH ^-/-^ / re-fed wild type)** | | | | |
| **Protein accession number** | **Peptide sequence** | **log10 Ratio** | **Protein name** | **Index** |
| IPI00125460 | K.GEM$DTFPTFK@FDDPKFEVIDKPQS.- | 1.49 | ATP synthase-coupling factor 6, mitochondrial | 15 |
| IPI00323592 | K.ITPFEEK@M$IAEAIPELK.A | 1.00 | Malate dehydrogenase, mitochondrial | 46 |
| IPI00127206 | K.DGVDFGK@WR.A | -1.06 | Fructose-bisphosphate aldolase B | 19 |
| IPI00226430 | R.FGTK@FGLDLK.L | -1.23 | 3-ketoacyl-CoA thiolase, mitochondrial | 30 |

IPI00466128 [R.GDNPFPK@NADGTVR.Y](mailto:R.GDNPFPK@NADGTVR.Y) -1.38 Alcohol dehydrogenase [NADP+] 49

|  |  |  |  |  |
| --- | --- | --- | --- | --- |
